# Attitudes, Opportunities, and Challenges for Clinical Pharmacy Services in Mizan-Tepi University Teaching Hospital, Southwest Ethiopia: Health Care Providers' Perspective

**DOI:** 10.1155/2020/5415290

**Published:** 2020-03-31

**Authors:** Solomon Hambisa, Abebaw Abie, Dejen Nureye, Mohammed Yimam

**Affiliations:** ^1^Department of Pharmacy, College of Medicine and Health Sciences, Mizan-Tepi University, Mizan-Teferi, Ethiopia; ^2^Department of Pharmacy, College of Medicine and Health Sciences, Dabre-Markos University, Dabre-Markos, Ethiopia

## Abstract

**Background:**

Clinical pharmacy is a branch of health sciences that focuses more on the patient than on drug product-oriented services to optimize drug therapy. This study aimed to assess attitudes, opportunities, and challenges for clinical pharmacy services from the health care providers' perspective in Mizan-Tepi University Teaching Hospital.

**Methods:**

A cross-sectional study was conducted among physicians, nurses, pharmacy professionals, and public health officers working in Mizan-Tepi University Teaching Hospital. A total of 119 health care providers participated in the study, and data were collected using a pretested self-administered questionnaire. The study tool was designed based on the instruments used in the previously conducted studies. Collected data were coded, entered, and analyzed using Statistical Package for the Social Sciences (SPSS, version 21). Furthermore, the descriptive and inferential statistics were performed.

**Results:**

Out of 119 health care providers included in the study, 59.66% of them were nurses. The majority of the health care providers (85.71%) had a positive attitude towards clinical pharmacy services. Most of the study participants mentioned that acceptance of clinical pharmacy services among health care providers as a major opportunity to clinical pharmacy services in Mizan-Tepi University Teaching Hospital. The major challenges described for the clinical pharmacy services include lack of support from hospital management, absence of clearly defined roles and responsibilities for the clinical pharmacists, and shortage of pharmacy workforce and staff turnover.

**Conclusion:**

Proper strategies should be in place to improve clinical pharmacy services and promote pharmacists' role in providing patient care.

## 1. Background

In contrast to the traditional pharmacy profession, clinical pharmacy is a branch of health sciences that focuses more on the patient than on drug product-oriented services to optimize drug therapy [1, 2]. Furthermore, this field of pharmacy practice is developed to enhance therapeutic benefits, reduce risk associated with medications and health care costs, and respect patients' preference [3–5].

Clinical pharmacy services incorporate a set of activities performed in the health care settings [6]. However, to deliver clinical pharmacy services, the presence of qualified and skilled clinical pharmacists is necessary in the existing health care system [7]. The main activities performed by a clinical pharmacist include, among others, interviewing patients about their medical conditions, formulating medication histories, providing recommendations for drug selection, and follow-up of drug therapy outcomes [4, 8]. Besides, the pharmacists should collaborate with other health care practitioners to foster quality of health care [8, 9].

Implementation of clinical pharmacy services and integration of pharmacists into the health care team plays a pivotal role in achieving a better pharmaceutical care and team decision-making [10]. Similarly, the evidence in the literature shows the impact of clinical pharmacy services in promoting pharmacotherapy, particularly in developing countries. However, there is disparity among countries regarding its implementation [11, 12]. In Ethiopia, many hospitals are providing clinical pharmacy services nowadays [13].

Several studies have been also conducted to assess health care providers' perception towards clinical pharmacy services. In Saudi Arabia, about 92.4% of the health care providers highlighted the importance of clinical pharmacists in the health care team [9]. In the United Arab Emirates, 74% of the respondent health professionals appreciated the role of clinical pharmacists in minimizing medication error and improving patient treatment outcomes [14]. In contrary, in Kuwait, 48.2% of physicians were found to be uncomfortable with a pharmacist's suggestion on the use of prescription medicines, and one-third of them were not expect the pharmacist to be available for consultation during rounds [15]. According to the study done in Ethiopia, more than 70% of the health care providers had a positive attitude towards clinical pharmacy services [5].

A plethora of studies have explored opportunities for the clinical pharmacists [10, 16–18]. In contrary, the clinical pharmacists are facing many challenges while dispensing medications, counseling patients, and interacting with medicine prescribers [17–21]. A recent reform in hospital implementation guidelines of Ethiopia stated the importance of pharmacists and their incorporation into the health care team for the benefit of the patients [22]. Likewise, a better understanding of health professionals' perception towards clinical pharmacy services can render a greater opportunity to identify the challenges and opportunities for the clinical pharmacists [17]. For this reason, this study attempted to assess attitudes, opportunities, and challenges towards clinical pharmacy services from health practitioners' perspective in Mizan-Tepi University Teaching Hospital (MTUTH).

## 2. Methods and Materials

### 2.1. Study Design and Sample Size

A cross-sectional study was conducted among physicians, nurses, pharmacy professionals, and public health officers working in MTUTH from February to March, 2017. Geographically, this hospital is located in Mizan-Aman town, southwest of Ethiopia. The hospital provides different inpatient and outpatient services for the communities living in the Mizan-Aman town and its surrounding area. It has a range of specialties including pediatrics, internal medicine, surgery, gynecology, and ambulatory care services. Likewise, the hospital has five different pharmacies such as outpatient pharmacy, antiretroviral therapy pharmacy, emergency pharmacy, and inpatient pharmacy to give pharmaceutical services for the patients. Recently, the clinical pharmacy service is initiated in different parts of Ethiopia. Accordingly, graduates in clinical-oriented pharmacy deployed in MTUTH to provide this service in various clinical sites such as ambulatory, internal medicine, surgery, pediatrics, and the drug information center.

At the time of study, the hospital has one hundred ninety-eight health care providers providing different health services. Furthermore, all health care providers who had a direct contact with the clinical pharmacy services and were available during the study period were included in the study. In connection to this, a total of 119 health care providers participated in the study. On the other hand, health care providers that were not willing to participate in the study were excluded from the study.

### 2.2. Data Collection Tool and Procedure

The data were collected using a pretested self-administered questionnaire. The study tool was designed based on the instruments used in the previously conducted studies [5, 16–18]. It included six questions to measure health care providers' attitudes towards clinical pharmacy services and statements pertain to the opportunities and challenges for clinical pharmacy services. The questionnaire also addressed basic demographic characteristics of the study participants. The study participants were asked to answer questions related to health care providers' attitudes using the options “yes” or “no.” Furthermore, the study respondents answered questions related to opportunities and challenges for the clinical pharmacy services based on the context of the study setting.

To ensure the validity of the study tool and its use in the study setting, a pilot study was conducted with seven health care providers. Minor modifications were made based on the feedback obtained from the participants in the pilot testing to improve clarity of some questions. The pilot study data were excluded from the study results. Two pharmacists were recruited to assist the data collection process. Completeness and consistency of collected data were checked by the supervisors.

### 2.3. Data Analysis

The collected data were coded, entered, and analyzed using Statistical Package for the Social Sciences (SPSS, version 21). The descriptive and inferential statistics were performed. Descriptive statistics were used to describe the characteristics of study participants, health care providers' attitudes, and opportunities and challenges for the clinical pharmacy services. Responding “yes” answer to three and more out of the six attitude items was considered as a positive attitude towards clinical pharmacy services. Bivariate logistic regression was done to see the association between a dependent variable (attitude) and demographic (age, sex, profession, and years of experience) variables. Level of significance was set at *P* < 0.05.

## 3. Result

Among physicians, nurses, pharmacy professionals, and public health officers working in the MTUTH, 119 of them participated in the study. Besides, a response rate of the study participants was close to 91%. As it can be seen from Table 1, from a total of the study respondents, 59.66% of them were nurses. More than half (53%) of the study respondents were males. The majority of the study respondents (74.79%) were found in the range of 25 to 34 years, and only 1.68% of participants were above 55 years. Regarding years of experience, 28.57%, 46.23%, and 25.2% of the study respondents served MTUTH for less than 3 years, 3 to 6 years, and greater than 6 years, respectively.

More than 85% of health care providers had a positive attitude towards clinical pharmacy services. Multivariate analysis was also performed to look for an association between the attitude of the health care providers and demographic (age, sex, profession, and experience) variables. Thus, attitude of health care providers was not significantly associated with their demographic variables (*P* value >0.05) (Table 2). Regarding attitude items, more than 80% of the study respondents appreciated the integration of clinical pharmacy services into the health care delivery system. Similarly, more than 88% of the study respondents agreed on the item “clinical pharmacy services are important in the Ethiopian health care system.”

About 84% of the study respondents agreed on the item “initiation of clinical pharmacy services can reduce adverse drug reaction.” Furthermore, more than 79% of the respondents agreed on the statement “initiation of clinical pharmacy services can reduce health care costs.” In addition, about 82% of the study participants appreciated the presence of clinical pharmacists in the hospital wards (Table 3).

Regarding opportunities for the clinical pharmacy services in MTUTH, the majority of the study respondents (83.19%) stated that acceptance of clinical services among health care providers is an opportunity for the clinical pharmacy services. Besides, more than 60% the study participants highlighted the presence of supporting partners such as hospital manager and hospital pharmacy department among opportunities for the clinical pharmacy services. Furthermore, more than half (56.30%) of them indicated the presence good working environment as an opportunity for the clinical pharmacy services (Table 4).

On the other hand, as it can be observed from Table 5, about 75% of the study participants described a lack of support from the hospital management as a challenge for clinical pharmacy services in the hospital. Similarly, around 70% of the study participants depicted a lack of clearly defined roles and responsibilities for the clinical pharmacists among the challenges for the clinical pharmacy services in MTUTH (Table 5). Additionally, about 60% of the study respondents mentioned that a shortage of pharmacy workforce and staff turnover are the challenges for the clinical pharmacy services in MTUTH. In contrary, only 1.68% of the study respondents demonstrated the disagreement among health care providers as a challenge for clinical pharmacy services (Table 5).

## 4. Discussion

This study assessed the attitudes of health care providers towards clinical pharmacy services and tried to extract opportunities and challenges for clinical pharmacy services in MTUTH. It was observed that the majority of health care providers had a positive attitude towards clinical pharmacy services. This is concordant with a report that came out from Ethiopia [5]. It was indicated that health care practitioners' attitudes towards clinical pharmacy services may promote or hinder the implementation of the clinical pharmacy services and the role of the clinical pharmacists in the health care setting [23]. As a result, it is very important for the responsible bodies to promote recently launched clinical pharmacy services and expand the role of pharmacists.

The present study also revealed that there is a strong belief among health care providers that integration of clinical pharmacy services to the existing health care system is important to improve potential patient care. Therefore, it would be better to make a further attempt to incorporate clinical pharmacy services into the exiting health care system. Furthermore, there is a substantial passion among health care providers for clinical pharmacy services. This necessitates additional training on the impact of clinical pharmacy services for the health care providers. Most of participated health care providers also agreed on the importance of clinical pharmacy services in the Ethiopian health care system. This is inline with the other study done in Ethiopia, in which 80% of the health professionals perceived that clinical pharmacy service initiation is important for the Ethiopian health care system [5].

Interestingly, most of the study respondents also believed that clinical pharmacy services are very helpful and important in reducing ADR and health care costs. This finding is also comparable with the studies done in Saudi Arabia [9] and the United Arab Emirates [14]. Thus, it is imperative that the concerned body should strive to strengthen and promote clinical pharmacy services. Similarly, a large proportion of health care providers appreciated the presence of clinical pharmacists in the hospital wards. For better pharmaceutical care; it would be better to assign more clinical pharmacists in the different clinical sites to minimize medication errors and improve the patient's quality of life.

The largest proportion of the study participants stated acceptance of clinical pharmacy services among the health care providers as a major opportunity for the clinical pharmacy services in MTUTH. This enables the clinical pharmacists to be exposed to a multidisciplinary health care team and thus practically render clinical pharmacy services. However, clinical pharmacists are expected to upgrade their therapeutic knowledge, experience, and skills which are used to achieve desired patient outcomes. Furthermore, this finding mirrors with the other study done in Ethiopia [17]. In addition, it was mentioned that presence of supporting partners such as hospital manager and hospital pharmacy department, better working environment, good team spirit among the health care providers, commitment among health care provider, and government policy towards patient centered pharmaceutical services as the other opportunities for the clinical pharmacy services in MTUTH. As a clinical pharmacy service is a recent incident in the Ethiopian health care system, these enable the services to be implemented at an advanced level.

On the other hand, this study indicated that lack of a support from hospital management, and lack of clearly defined roles and responsibilities for the clinical pharmacists are the major challenges for the clinical pharmacy services in MTUTH. A growing body of evidence demonstrated that without enough support from the management side, the service will not move forward to meet its purported goals [5]. To improve quality of health care in the hospitals, it is therefore imperative to use all available means to overcome the obstacles. Besides, a clearly defined scope of practices for all health professionals must be developed to fulfill the needs of the patients.

This study also reflected a shortage of pharmacy workforce and staff turnover, absence of a follow-up from responsible bodies, and a lack of enough salaries and incentives for health care providers as the challenges for the clinical pharmacy services in MTUTH. Similar findings were also reported in earlier studies conducted in Ethiopia [5, 24] and elsewhere in Asia [13]. Hence, the hospital leadership should look into the possibilities of recruiting more health care providers and training of more professionals to render better health services. In addition, responsible bodies should explore mechanisms to incentivize the pharmacists.

## 5. Conclusion

A large proportion of the study participants had a positive attitude for the clinical pharmacy services. Most of the study participants mentioned acceptance of clinical pharmacy services among health care providers, presence of supporting partners such as hospital manager and hospital pharmacy department, better working environment, and good team spirit among health care providers as the opportunities for the clinical pharmacy services. The major challenges for clinical pharmacy services described were a lack of support from hospital management, absence of clearly defined roles and responsibilities for the clinical pharmacists, a shortage of pharmacy workforce and staff turnover, a lack of follow-up from responsible bodies, and a lack of enough salaries and incentives for the health care providers. To strengthen and keep continuity of the clinical pharmacy services, the good side forwarded should be used wisely. Proper strategies should also be in place to improve clinical pharmacy services and promote the role of pharmacists in providing patient care.

## Figures and Tables

**Table 1:** Demographic profile of health care providers in MTUTH, February to March 2017.

Demographic information	Frequency (%)
*Gender*	
Male	63 (53)
Female	56 (47)

*Age distribution*	
<25	13 (10.92)
25–34	89 (74.79)
35 and above	17 (14.28)

*Profession*	
Nurse	71 (59.66)
Physician	25 (21)
Pharmacy professional	17 (14.28)
Public health officer	6 (5.04)

*Years of work experience in the health care setting*	
<3	34 (28.57)
3–6	55 (46.23)
>6	30 (25.2)

**Table 2:** Association of demographic characteristics with attitude of health care providers towards clinical pharmacy services in MTUTH, February to March 2017.

Demographic characteristics		Attitudes		
		Positive	Negative	AOR (95% CI)
Gender	Male	54	9	1.0
	Female	48	8	0.773 (0.235–2.540)^*∗*^

Age distribution	<25	11	2	0.996 (0.079–12.600)^*∗*^
	25–34	79	10	2.330 (0.440–12.328)^*∗*^
	35 and above	12	5	1.0

Profession	Physician	19	6	1.0
	Pharmacy professional	16	1	4.628 (0.471–45.488)^*∗*^
	Nurse	62	9	3.070 (0.723–13.046)^*∗*^
	Public health officer	6	1	2.325 (0.199–27.154)^*∗*^

Years of work experience in the health care setting	<3	31	3	2.286 (.297–17.616)^*∗*^
	3–6	47	8	1.088 (.217–5.460)^*∗*^
	>6	24	6	1.0

^*∗*^Statistically not significant adjusted odds ratio (AOR) (*P* value >0.05).

**Table 3:** Health care providers' attitudes towards clinical pharmacy services in MTUTH, February to March 2017.

Attitude items		Frequency (%)
Appreciating integration of clinical pharmacy services in the health care delivery system	Yes	98 (82.35)
	No	21 (17.65)
Having an interest to know more about clinical pharmacy services	Yes	106 (89.08)
	No	13 (10.92)
Clinical pharmacy services are necessary in the Ethiopian health care system	Yes	105 (88.24)
	No	14 (11.76)
Appreciating the presence of clinical pharmacists in the hospital wards	Yes	98 (82.35)
	No	21 (17.64)
Initiation of clinical pharmacy services can reduce adverse drug reaction	Yes	100 (84.03)
	No	19 (15.97)
Initiation of clinical pharmacy services can reduce health care costs	Yes	95 (79.83)
	No	24 (20.17)

**Table 4:** Opportunities for the clinical pharmacy services in MTUTH, February to March 2017.

Opportunities for the clinical pharmacy services	Frequency (%)
Acceptance of clinical pharmacy services among health care providers	99 (83.19)
Presence of supporting partners such as hospital manager and hospital pharmacy department	75 (63.03)
Presence of good working environment	67 (56.30)
Good team spirit among health care providers	60 (50.42)
Commitment of health care providers	55 (46.22)
Government policy towards patient-oriented pharmaceutical services	54 (45.38)

**Table 5:** Challenges for the clinical pharmacy services in MTUTH, February to March 2017.

Challenges for the clinical pharmacy services	Frequency (%)
Lack of a support from the hospital management	89 (74.79)
Lack of clearly defined roles and responsibilities for the clinical pharmacists	83 (69.75)
Shortage of pharmacy workforce and staff turnover	74 (62.18)
Lack of a follow-up from the responsible bodies	71 (59.6)
Lack of enough salaries and incentives	54 (45.38)
Lack of a support from pharmacy professionals	33 (27.73)
Disagreement among health care professionals	2 (1.68)
Lack of adequate knowledge of pharmacists to provide clinical pharmacy services	1 (0.84)

## Data Availability

The raw data used to support the findings of this study are available from the corresponding author upon request.
